# Comparison of Catecholamine Values Before and After Exercise-Induced Bronchospasm in Professional Cyclists

**Published:** 2017

**Authors:** Folly Messan, Albérick Tito, Polycarpe Gouthon, Kocou Basile Nouatin, Issiako Bio Nigan, Abel Sewanou Blagbo, Joseph Lounana, Jean Medelli

**Affiliations:** 1 Unité d’Exploration Respiratoire, Sport & Santé. Institut National de la Jeunesse, de l’Education Physique et du Sport (INJEPS), Université d’Abomey-Calavi (UAC), 01 BP 169 Porto-Novo, (Bénin).,; 2 Unité de Recherche : Performance Sportive, Santé et Evaluation. Institut National de la Jeunesse, de l’Education Physique et du Sport (INJEPS), Université d’Abomey-Calavi (UAC), 01 BP 169 Porto-Novo, (Bénin).,; 3 Unité Alimentation et Diététique du Sportif. Institut National de la Jeunesse, de l’Education Physique et du Sport (INJEPS), (INJEPS), Université d’Abomey-Calavi (UAC), 01 BP 169 Porto-Novo (Bénin).,; 4 Unité de Biologie de l’Effort et de Médecine du Sport ; CHU d’Amiens, Hôpital Nord (France)

**Keywords:** Adrenaline, Noradrenaline, Bronchospasm, Prevalence, Lung function, Cyclists

## Abstract

**Background:**

The concentration of circulating catecholamine increases during exercise in healthy athletes, but the variation has not been studied much in athletes who develop exercise-induced bronchospasm. This study measured changes in circulating catecholamine levels using the induced maximal effort test in the laboratory in professional cyclists sensitive to bronchospasm.

**Materials and Methods:**

This experimental study included 86 professional cyclists. They underwent two pulmonary function tests (to determine forced expiratory volume in one second [FEV_1_]) and two blood samples (to measure adrenaline and noradrenaline levels) were drawn before and after the stress test. Two subsets emerged: subjects whose FEV_1_ decreased by at least 10% from the resting value and non-sensitive subjects whose FEV_1_ do not meet this criterion.

**Results:**

A total of 51 cyclists (59%) were classified into the sensitive group. Resting catecholamine levels showed no significant difference (p > 0.05) between the two groups. In contrast, at the end of the exercise test, the adrenaline (581.9 ± 321.0 pg/mL versus 1783.5 ± 1001.0 pg/mL) and noradrenaline (4994.0 ± 2373.0 pg/mL versus 3205.0 ± 7714.4 pg/mL) levels were both lower in the sensitive group than those in the resting group (p < 0.0001).

**Conclusion:**

The frequency of the occurrence of bronchospasm observed in the studied cyclists was one of the highest among professional sports environments and the circulating catecholamine level was low in cyclists susceptible to bronchospasm. A training protocol adapted to their respiratory physiological profile may be indicated.

## INTRODUCTION

Catecholamine is derived from amino acids such as adrenaline and noradrenaline, which are also major components. Both hormones are synthesized by adrenal medulla cells (1, 2). Noradrenaline acts as both a neurotransmitter and a hormone, while adrenaline simply acts as a hormone. Catecholamine acts through membrane receptors (1) and at least two adrenergic receptor sites (α; β), which are divided into two groups: α_1_ and α_2_ receptors and β_1_, β_2_, and β_3_ receptors (3, 4). Noradrenaline activates α receptors and adrenaline acts on β receptors, although adrenaline can also activate α receptors (5). Among the sedentary and athletic, catecholamine concentrations are influenced by emotional factors and physical exercise among others. Indeed, exercise, hypoglycemia, and hypoxia are stressful situations that can cause instability in the major functions of organs (6). To restore homeostasis, the nervous and endocrine systems generate potent mediators involved in physiological regulation and, therefore, homeostasis. However, heightened secretion of catecholamine, including that of adrenaline and noradrenaline, occurs in response to exercise (6). Various studies have highlighted the role of catecholamine in the control of functions that affect physical performance and recent studies have focused on the effects induced by the duration and intensity of exercise on sympathoadrenal activity (7, 8). We also know that at a constant volume of oxygen consumption, continuous and substantial increases in the concentration of noradrenaline are observed in athletes (9–11). Even at very low-intensity exertion, increased adrenaline and noradrenaline concentrations are observed; however, the noradrenaline increase occurs faster than that of adrenaline (12). Increasing catecholamine concentrations are relatively more sensitive to high intensities that maximal aerobic power (13–22). For example, in training and in competition, professional cyclists are faced with high levels of intensity and exposure to air pollutants. Indeed, these cyclists are subject to a training volume of 30 hours per week and a maximum oxygen consumption of approximately 67 mL^−1^.kg^−1^. They travel more than 4,000 km of road per year and are most often exposed to air pollutants. The significant volume of air commonly ventilated by professional cyclists in adverse environmental and climatic conditions is likely to cause respiratory airway damage. Additionally, microcracks generated by hyperventilation cause cellular permeability to Na^+^, Cl^−^, K+, and Ca^2+^. They induce the release of chemical mediators involved in the initial inflammatory process-induced bronchospasm of exercise. Hyperventilation in a polluted atmosphere is detrimental to the lung function of these cyclists, leading to ventilator disorders and an increased asthma prevalence from 48 % to 55 % has been reported in athletes (23–28). Physical exercise and sports training impose stress to the central nervous system that subsequently triggers two feedback loops in the hypothalamus and the adrenal medulla, generating acetylcholine and catecholamine, respectively. Catecholamine contributes to smooth muscle relaxation and inhibition of the release of mediators of inflammation by binding to mast cell receptors, preventing their bridging. As exercise-induced bronchospasm (EIB) is characterized by respiratory airway resistance and the flow of inspired air, it is reasonable to hypothesize that, in susceptible athletes with exercise-induced bronchospasm, the concentration of catecholamine at the end of physical exertion is lower than that in non-sensitive athletes. Therefore, to test this hypothesis, this study was conducted among professional cyclists with the following aims: 1) diagnose EIB and 2) compare the postexercise concentrations of catecholamine in subjects who are sensitive and non-sensitive to EIB.

## MATERIALS AND METHODS

### Experimental Design

The study sample comprised 86 male professional cyclists aged 19 to 33 years whose anthropometric characteristics are presented in Table 1. Under the mandatory medical monitoring imposed by the French Cycling Federation, successful athletes were evaluated at the University Hospital Center of Amiens (France) approved by the Federation. After freely providing their informed and written consent, the athletes were familiarized with the protocols and equipment. Upon their arrival at the laboratory starting at 8:30 a.m., they were weighed and their heights were measured by conventional methods. To eliminate any medical indication against the maximal exercise test, the subjects were all subjected to a complete physical examination and a resting electrocardiogram. They were previously asked to refrain from strenuous physical effort during the 24 hours before the test, the consumption of stimulants (coffee, tobacco, and alcohol), or medication that could influence the measured parameters. They also had to eat their last meal at least three hours before the test.

**Table 1.** Comparing anthropometric data, noradrenaline, adrenaline mean values observed before and after exercise between EIB (+) and EIB (−) groups (mean ± standard deviation).

	**EIB (+) (n = 51)**	**EIB (−) (n = 35)**	**Difference**
**Height** (cm)	179.18 ± 5.09	181.34 ± 6.19	ns
**Weight** (kg)	70.10 ± 4.80	71.22 ± 5.80	ns
**Age** (yr)	25.16 ± 3.00	26.00 ± 3.66	ns
**NA/before** (pg/mL)	391.00 ± 183.00	391.74 ± 174.00	ns
**NA/after** (pg/mL)	4994.03 ± 2373.00	7714.42 ± 3205.00	p < 0.001
**A/befor**e (pg/mL)	79.88 ± 59.00	58.91 ± 51.00	ns
**A/after** (pg/mL)	581.92 ± 321.00	1783.51 ± 1001.00	p < 0.001

EIB (+): Subjects with bronchospasm; EIB (−): Subjects without bronchospasm; NA: noradrenaline; A: adrenaline; pg: picogram; p < 0.001: difference significant at p < 0.001; ns: no significant; the two ways ANOVA (EIB status × time of measurement) was significant for both NA (F = 22.6; p < 0.001) and A (F = 62.3; p < 0.001); Delta value (%) for A/after – A/before was + 7.10^2^ within EIB (+) and + 30.10^2^ within EIB (−) ; Delta Value (%) for NA/after –NA/before was + 13.10^2^ within EIB (+) and + 20.10^2^ within EIB (−).

### Blood sampling and stress test

The laboratory nurse established venous access to the forearm of each subject. A sterile 18 G catheter was connected for the infusion (Perfupack) of 500 mL sodium chloride. A three-way valve with injection valve and 25-cm extension allowed blood sample sterility without painful embarrassment or hindrance to the actions of the subject. The isotonic saline solution was used to rinse and keep track of blood access. The stress test was performed on a cycle ergometer (Lode Excalibur Sport). The ergometer seat, handlebars, and frame were adjustable to adapt to the morphology of each subject. The stress test was triangular; the subjects were exposed to a power increment of 50 Watts every three minutes until exhaustion. The inability of the subject to maintain a cadence of 90 rev/min indicated fatigue.

### Bronchospasm assessment test

Each subject, standing with a pinched nose, was instructed to breathe calmly and naturally into the mouthpiece connected to the computer-calibrated pneumotachograph spirometer device (Master lab, Jaeger, Strasbourg, France). Each was then instructed to inspire maximally to fill his lungs with air and to then expel the air continuously and completely for at least six seconds. After the test, the best result was selected from among the three measurements which were validated according to the spirometer algorithms. Systolic and diastolic blood pressures were monitored continuously during each exercise test because the monitoring of these parameters is imperative in France during exercise testing.

### Measurement of catecholamine levels

The noradrenaline and adrenaline concentrations were measured by amperometry extraction after separation by high-performance liquid chromatography (HPLC). The HPLC equipment used in this study included: 1) an automatic sample injection system (WatersTM 717 plus Autosampler); 2) a reservoir containing the mobile phase consisting of sodium acetate, citric acid, octane sulfonate, di-N-butylamine, EDTA, and methanol; 3) a pump (Waters model 510 HPLC pump); 4) a pre-column dolly; 5) an electrochemical detector (Waters 460 Electrochemical Detector); and 6) an integrating recorder (Waters Data Module). Blood samples were collected in a Monovette EDTA tube that was centrifuged at 3,000 revolutions/minute at 1,400 × g; the plasma was frozen and stored at −80 °C. The thawed and pretreated samples were then subjected to chromatography. After three minutes of centrifugation at 5,000 rpm, the supernatant of approximately 90 μL containing catecholamine and the internal standard were injected into the HPLC system for analysis lasting approximately 16 minutes per sample. Catecholamine and the internal standard were eluted from the column in the following order: the first peak corresponded to noradrenaline, the second to adrenaline, and the final two to Dihydroxybenzylamine (DHBA) and dopamine, respectively. Based on comparisons of the areas of each peak with that of DHBA (standard) with a known concentration, the calculated integrator was used to determine the concentrations of noradrenaline and adrenaline.

### Study variables

The bronchospasm test was considered positive and diagnosis made if the postexercise expiratory volume (FEV_1_) was decreased by at least 10% from the resting value, based on the formula: ΔVEMS (%) = (postexercise FEV_1_ × 100) / (resting FEV_1_) − 100. The status of each cyclist in connection to his EIB sensitivity (EIB + or −) was the first independent variable and the time of measurement (pre-test and post-test) was the second. The concentrations of noradrenaline, adrenaline, as dependent variables, expressed as picogram per milliliter (pg/mL), were determined based on the peak area and compared with known DHBA concentrations.

### Statistical analysis

The anthropometric values of the EIB (+) and EIB (−) groups were compared using unpaired Student’s t-tests. Similarly, the catecholamine concentrations observed at rest and at late efforts were compared to each modality between the two groups using a two-way analysis of variance (ANOVA) (for interaction EIB status × time of measurement), followed by unpaired Student’s t-tests. The level of significance of the statistical tests was set at p< 0.05.

## RESULTS

### Features of the studied cyclists

The characteristics of the subjects are presented in Table 1. At the beginning of the study, the age, height, and body weight did not differ significantly between the EIB (+) and EIB (−) groups. Comparisons of the catecholamine values according to cyclists’ status based on a 10% reduction in FEV_1_ revealed that 51 of 86 cyclists met this criterion (a prevalence of 59%). The EIB of the FEV_1_-sensitive cyclist group was reduced by an average of 12.71% while that of the non-sensitive group improved by an average of 10.3%. There was an interaction (EIB status × time of measurement), since the result of the two-way ANOVA was significant (p < 0.0001). From the idle state to the end of the exercise test, the average adrenaline concentration increased by 7.10^2^ and 30.10^2^% in the EIB-sensitive and non-sensitive cyclist group, respectively. From the idle state to the end of the exercise test, the average noradrenaline concentration increased significantly by 13.10^2^ and 20.10^2^% the EIB-sensitive and non-sensitive groups of cyclists, respectively (p < 0.0001 for both).

## DISCUSSION

This study determined the variation in catecholamine concentrations, particularly those of adrenaline and noradrenaline, among professional cyclists sensitive to bronchospasm. The bronchial provocation test performed using a stress test revealed an asthma prevalence of 59% in these cyclists. This value is close to that previously reported in professional road cyclists (52%) (23). Similarly, Rundell et al. (27) assessed elite athletes, reporting a prevalence of 51%, while Mannix et al. (28) reported a prevalence of 55% in gliding sports. The observed high prevalence of bronchospasm (59%) may have several explanations: professional cyclists annually traverse 4,000 km, have a training schedule of 10 to 12 hours per week and travel 60 to 100 km for competition. Similarly, these cyclists participate in 35 to 40 competitions annually. The intensity levels of these competitions are high and cyclists are forced to expire at a VO_2max_ which borders and sometimes exceeds 70 mL.kg^−1^.min^−1^. The significant volume of air inhaled by these professional cyclists well proves the need for hyperventilation associated with workouts and competitions. This leads to increased Ca^2+^, K^+^, Cl^−^, and Na^+^ levels and mediators of inflammation are released to facilitate the abrupt contraction of the smooth muscles of the airways (29,30). Physical exercise leads to this process, triggering bronchial spasms in these cyclists, proving that this phenomenon is from exercise-induced bronchospasm. In fact, exercise is a stress that causes an imbalance in the major functions of the body of the athlete. Therefore, the secretion of hormones is necessary to restore the balance of these functional systems. Thus, the nervous and endocrine systems generate very powerful mediators such as adrenaline and noradrenaline whose concentrations increase depending on the duration, intensity, and type of exercise. For submaximal dynamic exercise, the duration of exercise plays a key role in the adrenergic activity (31). Moussa et al. (16) observed a significant increase in catecholamine after a sprint run on an ergocycle for 30 and 6 seconds at constant load in untrained subjects. Moreover, Caillaud et al. (32) showed that a minimal exercise time was required to induce an increase in catecholamine, even at high intensity. However, this hypothesis has not been not confirmed because the study methods differ from each other (16, 22, 33). In fact, the results of the present work suggest that adrenaline and noradrenaline concentrations are more sensitive to exercise intensity (34–36). Similarly, noradrenaline concentration increases exponentially with exercise intensity (37, 38). Kajaer et al. (39) showed that plasma noradrenaline is sensitive to an increase after 60 minutes of exercise when performed at 35% of VO_2max_ compared to 20 minutes of exercise performed at 40–50% of VO_2max_ and that the increase occurs prior to an increase in adrenaline concentration. Based on work by Zouhal (6), the sensitivity to increased plasma concentrations of adrenaline and noradrenaline, although linked to a rise in VO_2max_ and heart rate, seems to be higher after a year of isometric and dynamic exercise. Similarly, the time duration that the subjects are observed prior to blood sampling may be a variable factor. For example, after brief and intense exercise, Hagberg et al. (40) showed that the concentrations of catecholamine quickly return to their baseline values and even fall by 35% after one minute of rest (38, 41). Therefore, in this study, blood samples were taken immediately after exercise testing among professional cyclists. Indeed, when the waiting period between exercise end and blood sampling exceeds three minutes, the catecholamine concentration levels drop considerably because this time is higher than the half-life of these amino acids. In summary, studies have observed increased catecholamine concentrations depending on the duration, intensity, and type of exercise. However, the status of catecholamine is not yet demonstrated in professional cyclists sensitive to exercise-induced bronchospasm. The results of our study revealed an elevated plasma concentration of catecholamine. Compared to their initial concentrations observed in the resting state, the values of catecholamine recorded at the end of the exercise test showed increases of 7.10^2^% for EIB (+) and 30.10^2^% for EIB (−) in adrenaline and 13.10^2^% for EIB (+) and 20.10^2^% for EIB (−) in noradrenaline (Table 1). Therefore, there was a difference in the magnitude of the response by these biological parameters after exercise according to bronchospasm sensitivity in these subjects. Indeed, the resting state concentrations of adrenaline and noradrenaline observed the year before showed no significant difference between the bronchospasm sensitive and non-sensitive groups. Similarly, comparison of the anthropometric parameters between the two groups showed no significant difference (Table 1). In contrast, at the end of the exercise test, the concentrations of adrenaline and noradrenaline recorded in cyclists sensitive to bronchospasm were significantly lower than those observed in non-sensitive subjects. What mechanisms may explain the decreased catecholamine levels observed in sensitive individuals at the end of maximal effort, when both groups had early similar resting levels? In the non-sensitive cycling group, the stimulation of beta adrenergic receptor, the stable behaviors of mast cell degranulation and mediators in very small quantities are likely to be the cause of the observed responses and could signal the existence of mild bronchiectasis. The change in FEV_1_ observed in the non-sensitive group could induce catecholamine synthesis and catecholamine circulation after the stress test. Rundell et al. (27) proposed that excessively high intensities of exercise are likely to cause excessive catecholamine release. Conversely, in cyclists sensitive to bronchospasm, dehydrated epithelial cells, mast cell activation, and increased intracellular calcium concentrations are prerequisites for the abundant degranulation of inflammatory mediators. These successive phenomena can induce acute respiratory distress in the airways (42). Excessive circulating catecholamine may be released because of these very high intensities. They induce significant dilatation of the bronchi and vessels, thereby inhibiting the release of inflammatory mediators (43). Finally, they can bind mast cell receptors to prevent the stimulation and entry of calcium into the cell nucleus. Indeed, the adrenal hormone adrenaline is a powerful bronchodilator that protects against bronchoconstriction induced by histamine and prostaglandins. Noradrenaline reduces contractions of cholinergic origin at low concentrations, which does not affect the response to acetylcholine. Noradrenaline induces the inhibition of the release of acetylcholine at the neuromuscular synapse. This mechanism would be needed in cyclists susceptible to bronchospasm who have high levels of adrenaline and noradrenaline. Under these circumstances, it is clear that the level of catecholamine is lower at the end of effort in sensitive individuals than in non-sensitive subjects.

## CONCLUSION

Exercise induces increased catecholamine concentrations in athletes. This increase varies depending on the duration, intensity, and type of exercise. Adrenaline and noradrenaline are the main hormones whose levels increase substantially during exercise. The adrenal hormone adrenaline is a powerful bronchodilator that protects against histamine and prostaglandin-induced bronchospasm. In contrast, noradrenaline reduces contractions of cholinergic origin at concentrations that inhibit the effect of acetylcholine. Circulating catecholamines are recruited to mitigate the effect of bronchospasm on respiratory function in the airways of athletes. Thus, athletes that are sensitive to bronchospasm have relatively lower catecholamine concentrations after exercise or competition than those in non-sensitive subjects. Under these circumstances, the development of a training protocol adapted to their physiological respiratory profile would be appropriate.
